# IFI30 expression is an independent unfavourable prognostic factor in glioma

**DOI:** 10.1111/jcmm.15758

**Published:** 2020-09-23

**Authors:** Xiu Liu, Chunyan Song, Shoubo Yang, Qiang Ji, Feng Chen, Wenbin Li

**Affiliations:** ^1^ Beijing Tiantan Hospital Capital Medical University Beijing China; ^2^ Department of Neuro‐Oncology, Neurosurgery Center Beijing Tiantan Hospital Capital Medical University Beijing China

**Keywords:** glioma, *IFI30*, immune phenotype, prognostic factor

## Abstract

Gamma‐interferon‐inducible lysosomal thiol reductase, the only known lysosomal thiol reductase, is encoded by gene *IFI30* and expressed constitutively in antigen‐presenting cells. Our comprehensive study on *IFI30* in gliomas found its expression to be high in glioblastomas and in gliomas with a mesenchymal subtype or wild‐type isocitrate dehydrogenase, all of which indicated the malignancy and poor outcomes of gliomas. Kaplan‐Meier survival analysis ascertained that high *IFI30* expression conferred poor outcomes. The *IFI30* expression levels also showed high efficiency in predicting 1‐, 3‐ and 5‐year overall survival. Univariable and multivariable Cox regression analyses were performed to define *IFI30* as an independent prognostic marker. Biological process analysis suggested that *IFI30* was involved in immune responses. ESTIMATE and CIBERSORT were applied to evaluate immune cell infiltration, with results indicating that samples with higher *IFI30* expression had higher infiltration of immune cells, including regulatory T cells and M0 macrophages. Correlation analysis showed that *IFI30* was significantly positively correlated with immune checkpoints that suppress effective antitumour immune responses. Immunohistochemical staining was also performed to confirm the association between *IFI30* expression and the immune phenotype. The suggested correlation between high *IFI30* expression and an immunosuppressive phenotype contributes to our knowledge about the glioma microenvironment and might provide clues for the development of novel therapeutic targets.

## 1 INTRODUCTION

Although scientists have been trying to activate the antitumoural potential of the immune system, the first‐line treatment of tumours is still limited to surgery, radiotherapy and chemotherapy. The recent successes of immune checkpoint inhibitors and chimeric antigen receptor T cell therapy in many cancers underscore the prospects of immunotherapy and the importance of immunological interpretation.^1^, ^2^, ^3^, ^4^ An immune response that can effectively kill cancer cells involves a series of steps. First, tumour antigens (which differentiate tumour cells from normal cells) are captured and processed by dendritic cells. Second, the captured antigens are presented to T cells through major histocompatibility complex (MHC) I and II molecules, resulting in T cell priming and activation. Finally, the activated T cells migrate to and infiltrate the tumour where they recognize and kill the tumour cells.^5^, ^6^, ^7^ However, tumours have adopted multiple strategies to attenuate the attack of the immune system, ranging from the down‐regulation of immunogenic antigens and antigen‐presenting cells (APCs), to the prevention of T cell infiltration via vasculature barrier and through the suppression of effector T cells.^8^, ^9^ A deeper understanding of the mechanisms by which tumours escape immune attacks would facilitate innovative therapeutic strategies. In addition to immune checkpoint inhibitors and chimeric antigen receptor T cell therapy, a growing number of immunotherapeutics are under clinical investigation for their safety and efficacy, including peptide vaccines, dendritic cell vaccines, and therapies targeting chemokines in the tumour microenvironment.^10^, ^11^, ^12^, ^13^, ^14^

Gliomas are the most common type of primary malignant tumours of the central nervous system (CNS). With the substantial advance in the 2016 World Health Organization (WHO) classification of tumours of the CNS, the refinement of glioma classification on the basis of molecular biomarkers has led to a massive increase in the development of targeted therapies against this disease.^15^, ^16^, ^17^, ^18^ At the same time, persistent efforts have been made to reveal the immune characteristics of gliomas for the design of novel immunotherapeutic approaches. Immune cells, such as microglia, peripheral macrophages, leukocytes, and myeloid‐derived suppressor cells, are reported to infiltrate gliomas wherein they create an immunosuppressive microenvironment to facilitate tumour cell growth and invasion and to dampen the efficacy of immunotherapy.^19^, ^20^, ^21^ For example, tumour‐associated macrophages (TAMs) secrete cytokines such as transforming growth factor‐beta (TGF‐β) and interleukin‐10 (IL‐10) to inhibit effector T cells. TAMs express ligand receptors for programmed cell death protein 1 (PD‐1) and cytotoxic T‐lymphocyte antigen 4 (CTLA‐4) to suppress the cytotoxic functions of T cells.^22^, ^23^, ^24^ Hence, to achieve a breakthrough in glioma immunotherapy, a detailed understanding of the specific immune system is needed.

It was reported in a recent study that gamma‐interferon‐inducible lysosomal thiol reductase (GILT) is overexpressed in gliomas, and knockdown of the enzyme suppresses glioma cell proliferation through the promotion of apoptosis and induction of cell cycle arrest.^25^ GILT, encoded by the *IFI30* gene, is the only known lysosomal thiol reductase. It is constitutively expressed in APCs, including dendritic cells, monocytes/macrophages, and B cells. Interferon‐gamma can induce the expression of GILT in other cell types, such as melanoma cell lines. GILT catalyses disulfide bond reduction and enhances the MHC II‐restricted presentation of a subset of epitopes.^26^, ^27^, ^28^ GILT‐free mice were reported to be defective in antigen processing.^29^ In this study, we systematically analysed *IFI30* in 921 glioma samples sourced from the Chinese Glioma Genome Atlas (CGGA) and The Cancer Genome Atlas (TCGA) databases, including its expression in different tumour grades and subtypes, potential biological functions, and prognostic significance. We also evaluated the correlation between *IFI30* expression and some important immune‐related molecules. Immunostaining was performed to confirm the expression pattern of *IFI30* and its correlation with immune‐related factors. The results suggested that *IFI30* was a novel independent prognostic factor with immune‐related functions.

## 2 MATERIALS AND METHODS

### 2.1 Patients and data collection

In total, 310 CGGA‐sourced samples (105 grade II, 67 grade III and 138 grade IV gliomas, http://www.cgga.org.cn) and 611 TCGA‐sourced samples(214 grade II, 237 grade III, and 160 grade IV gliomas, http://cancergenome.nih.gov/) were studied. All analyses were performed using the CGGA data set and then validated with the cohort from TCGA. The clinical and molecular features of the patients are given in Table S1.

### 2.2 Immunohistochemical staining

Sections (5µm thick) of formalin‐fixed, paraffin‐embedded glioma tissues were deparaffinized and rehydrated and then incubated with Tris‐EDTA buffer (pH 9.0) for antigen retrieval. Thereafter, the tissue samples were incubated with primary antibodies for 2 hours at ambient temperature (anti‐IFI30 antibody, 1:10 000 dilution, Invitrogen, Carlsbad, CA, USA; anti‐CD163 antibody, 1:200 dilution, Abcam, Cambridge, UK; anti‐PD‐L2 antibody, 1:200 dilution, Proteintech, Rosemont, IL, USA; and anti‐IL‐10 antibody, 1:200 dilution, Proteintech). Then, the sections were rinsed, incubated with appropriate secondary antibodies (ZSGB‐Bio, Beijing, China), treated with 3,3′‐diaminobenzidine staining solution, and counterstained with Mayer's haematoxylin. The staining results were reviewed independently by two investigators.

### 2.3 Statistical analysis

Student's t test was used to determine differences between two groups. Kaplan‐Meier survival analysis and the log‐rank test were performed to assess the significance of *IFI30* expression to survival. Time‐dependent receiver operating characteristic (ROC) curve analysis was applied to evaluate 1‐, 3‐ and 5‐year overall survival (OS) prediction. Cox regression analysis was used to assess the prognostic value of *IFI30*. Pearson's correlation analysis was conducted to calculate the correlation between *IFI30* and other genes. DAVID Bioinformatics Resources 6.8 (https://david.ncifcrf.gov/) was used for Gene Ontology (GO) analysis. The ESTIMATE package and CIBERSORT (https://cibersort.stanford.edu/) were applied to evaluate the immune score and immune cell infiltration, respectively. All statistical analyses and graph generation were conducted with SPSS 23.0 (IBM, Armonk, NY, USA) and R software (R version 3.5.3; https://www.r‐project.org/). A p value of less than 0.05 was considered to be statistically significant.

## 3 RESULTS

### 3.1 *IFI30* expression was up‐regulated in glioblastomas, and in gliomas with wild‐type isocitrate dehydrogenase and mesenchymal subtype

**Figure 1:**
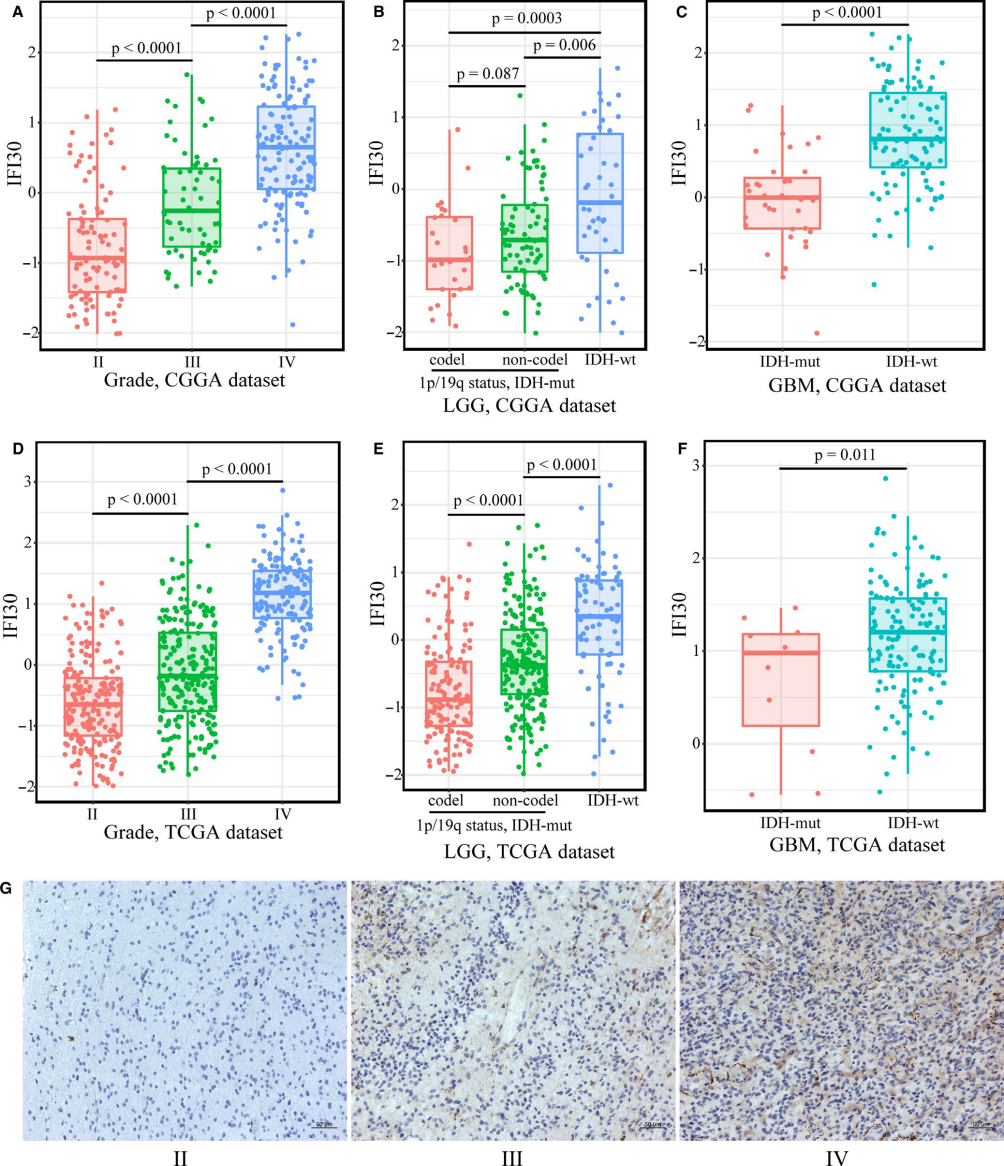
*IFI30* expression in stratified gliomas. (A and D) *IFI30* expression in different tumour grades in the CGGA and TCGA data sets. (B and E) *IFI30* expression in LGG stratified according to IDH mutation status and 1p/19q codeletion status in the CGGA and TCGA data sets. (C and F) *IFI30* expression in GBM with mutant or wild‐type IDH in the CGGA and TCGA data sets. (G) Representative images of *IFI30* immunostaining in different grades of glioma samples (bar, 50µm)

To clarify the characteristics of *IFI30* in gliomas, we first analysed its expression level in the CGGA and TCGA data sets stratified according to the tumour grade, isocitrate dehydrogenase (IDH) mutation status, and 1p/19q codeletion status. As shown in Figure 1A,D, *IFI30* expression was significantly increased along with the grade of the tumour and was the highest in glioblastomas (GBM, glioma grade IV). In the gliomas that were stratified on the basis of the IDH and 1p/19q status, the *IFI30* expression level was the highest in the IDH wild‐type (IDH‐wt) group, both in the lower‐grade gliomas (LGG, grade II and III) and GBM (Figure 1B,C for CGGA, Figure 1E,F for TCGA). The *IFI30* expression level in different grades of gliomas was also confirmed by immunohistochemical staining (Figure 1G). These results indicated that a high level of *IFI30* expression implied malignant progression of the glioma.^30^, ^31^, ^32^

**Figure 2:**
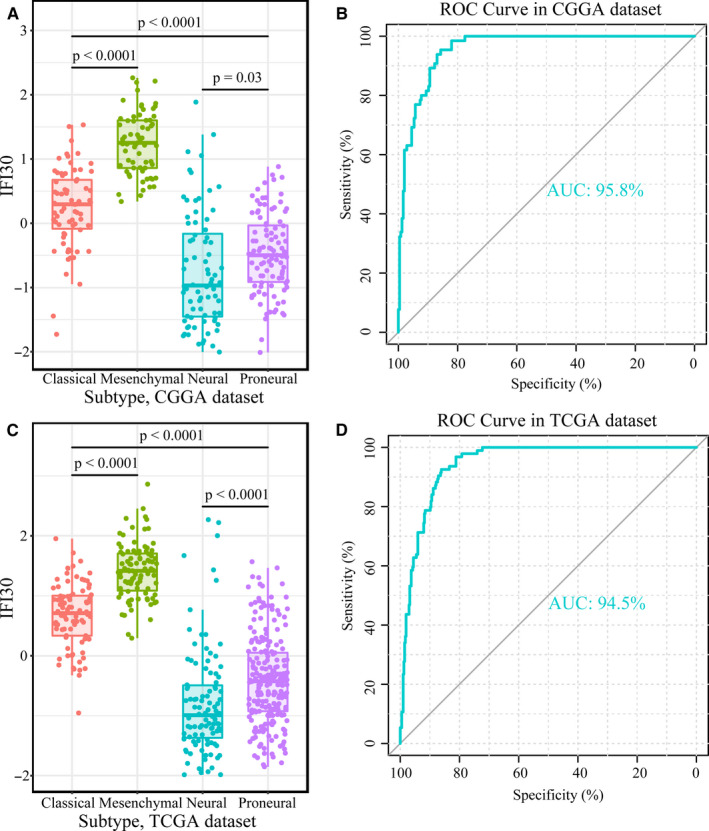
(A and C) *IFI30* expression in different molecular subtypes in the CGGA and TCGA data sets. (B) ROC curve analysis of the efficiency of *IFI30* expression in predicting the mesenchymal subtype in the CGGA data set, with a specificity of 0.857 and a sensitivity of 0.954. (D) ROC curve analysis of the efficiency of *IFI30* expression in predicting the mesenchymal subtype in TCGA data set, with a specificity of 0.861 and a sensitivity of 0.926

Next, we explored the distribution of *IFI30* in four TCGA molecular subtypes. In both the CGGA and TCGA data sets, the mesenchymal subtype showed the strongest expression of *IFI30*, followed by the classical, proneural, and neural subtypes successively (Figure 2A,C). The gliomas were next divided into mesenchymal and non‐mesenchymal subtype groups for ROC curve analysis, which showed the high efficiency of the *IFI30* expression level in predicting the mesenchymal subtype. This was further suggested by the area under the ROC curve (AUC), which was 0.958 and 0.945 for the CGGA and TCGA cohort, respectively(Figure 2B,D). All the results demonstrated the association between *IFI30* expression and the malignant phenotype of gliomas.

### 3.2 High *IFI30* expression correlated with poor outcomes and was an independent prognostic predictor

**Figure 3:**
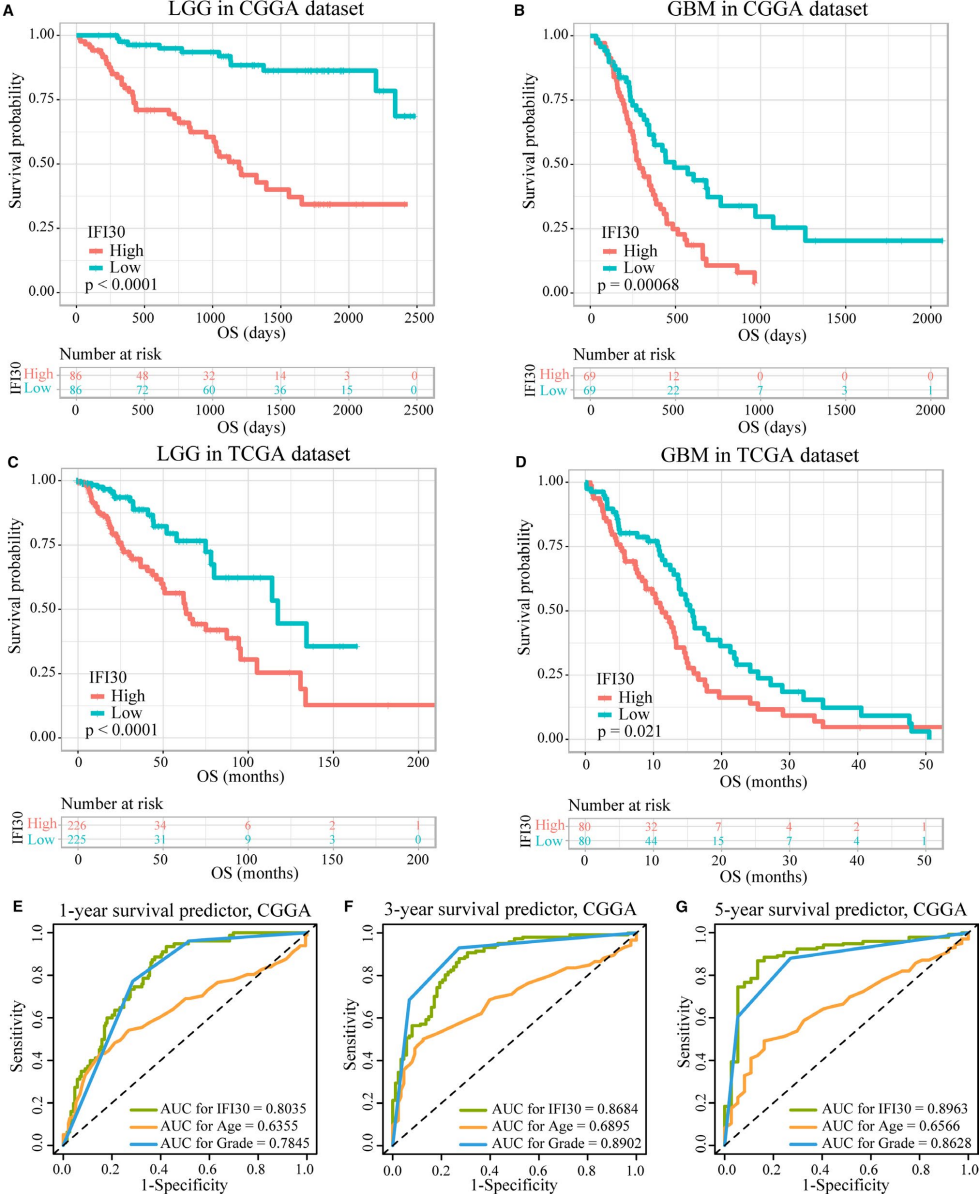
Survival analysis of *IFI30* in gliomas. (A‐D) Kaplan‐Meier survival analysis of LGG and GBM in the CGGA and TCGA data sets. (E) Time‐dependent ROC curve analysis of the efficiency of *IFI30* expression, patient age, and tumour grade in predicting 1‐year OS in the CGGA data set. The specificity and sensitivity were 0.577 and 0.935 for *IFI30*, 0.847 and 0.433 for age, and 0.714 and 0.773 for grade, respectively. (F) Time‐dependent ROC curve analysis of the efficiency of *IFI30*, age and grade in predicting 3‐year OS in the CGGA data set. The specificity and sensitivity were 0.727 and 0.880 for *IFI30*, 0.875 and 0.501 for age, and 0.727 and 0.930 for grade, respectively. (G) Time‐dependent ROC curve analysis of the efficiency of *IFI30*, age and grade in predicting 5‐year OS in the CGGA data set. The specificity and sensitivity were 0.865 and 0.868 for *IFI30*, 0.838 and 0.491 for age, and 0.730 and 0.880 for grade, respectively

Next, we evaluated the prognostic value of the*IFI30* expression level. Kaplan‐Meier survival analyses were performed separately on the LGG and GBM, with the median *IFI30* expression level used as a cut‐off. In both the patients with LGG and those with GBM, high *IFI30* expression was associated with decreased OS. Consistent results were obtained with both the CGGA and TCGA data sets (Figure 3A‐D). We further stratified the patients on the basis of IDH mutation and 1p/19q codeletion status. For the patients with LGG with IDH‐wt, a higher level of *IFI30* expression was associated with a shorter OS in both the CGGA (Figure S1C) and TCGA (Figure S2C) cohorts. In addition, the *IFI30* expression level could distinguish the prognosis of patients with LGG with a mutant IDH (IDH‐mut) and non‐codeleted 1p/19q (1p/19q non‐codel) in the data set from the CGGA (Figure S1A), and patients with GBM with IDH‐wt in the data set from TCGA (Figure S2E). Next, we compared the specificity and sensitivity of *IFI30* expression, the patient age at diagnosis, and the tumour grade in predicting OS. The AUCs based on *IFI30* expression for 1‐, 3‐, and 5‐year OS were 0.8035, 0.8684, and 0.8963, respectively, in the CGGA data set and were larger than the corresponding AUCs based on age and grade, except for the AUC based on the tumour grade for 3‐year OS (Figure 3E‐G). In the data set from TCGA, although the AUC based on *IFI30* expression for 5‐year OS was smaller than that based on age and grade, *IFI30* expression was still a better predictor of 1‐year OS compared with grade, and of 3‐year OS compared with age (Figure S3). These results demonstrated that a high *IFI30* expression conferred poor outcomes in patients with gliomas and that the gene expression level could predict OS effectively.

**Figure 4:**
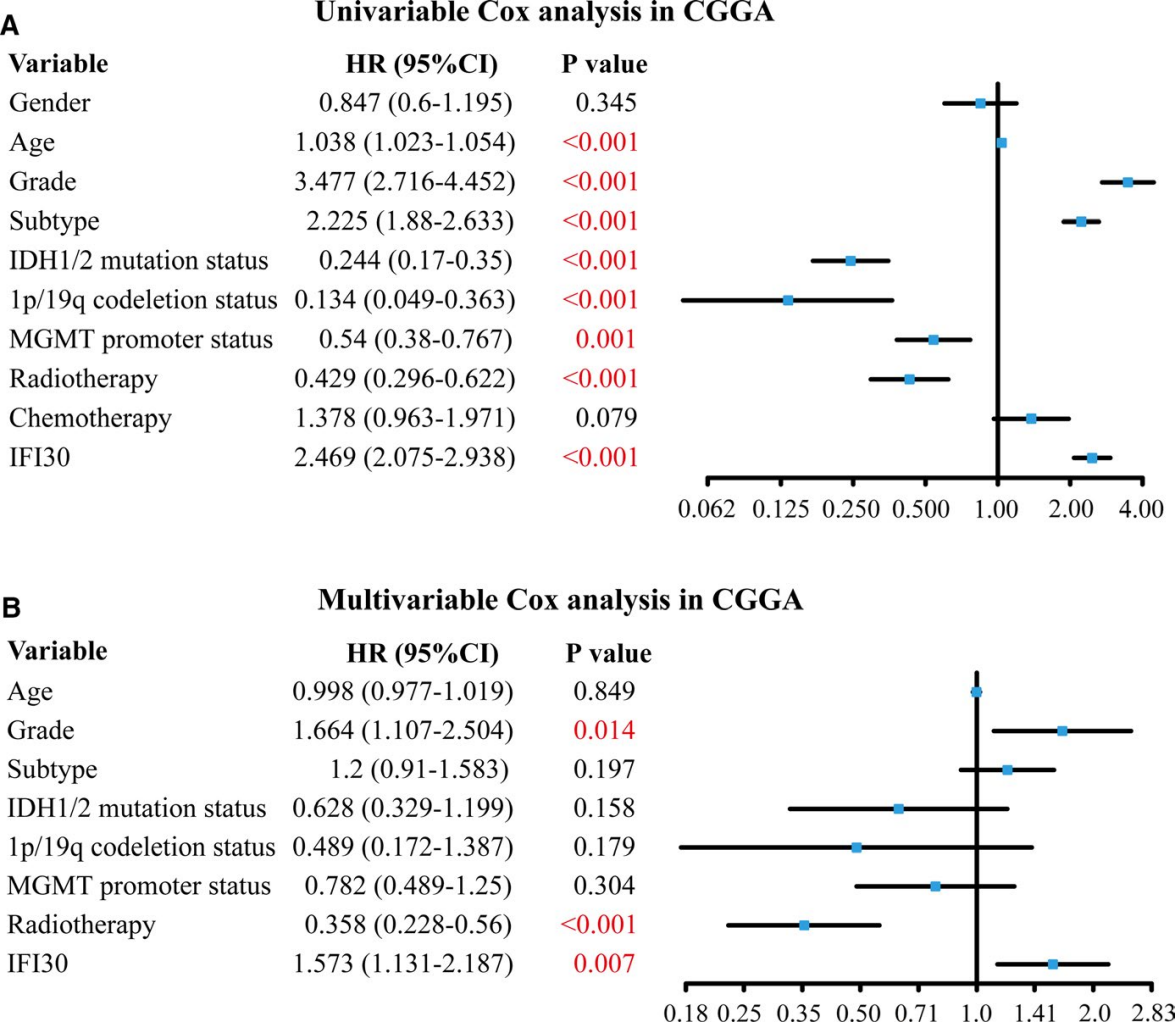
Univariable and multivariable Cox regression analyses of *IFI30* expression and several other clinical factors in the CGGA data set

Furthermore, we conducted univariable and multivariable analyses to confirm whether *IFI30* expression was an independent prognostic biomarker for gliomas. As shown in Figure 4, *IFI30* expression was still significantly associated with patient survival in the CGGA data set after multivariable Cox regression analysis, a characteristic that was validated with the cohort from TCGA (Figure S4).

### 3.3 *IFI30* expression was associated with immune‐related functions

**Figure 5:**
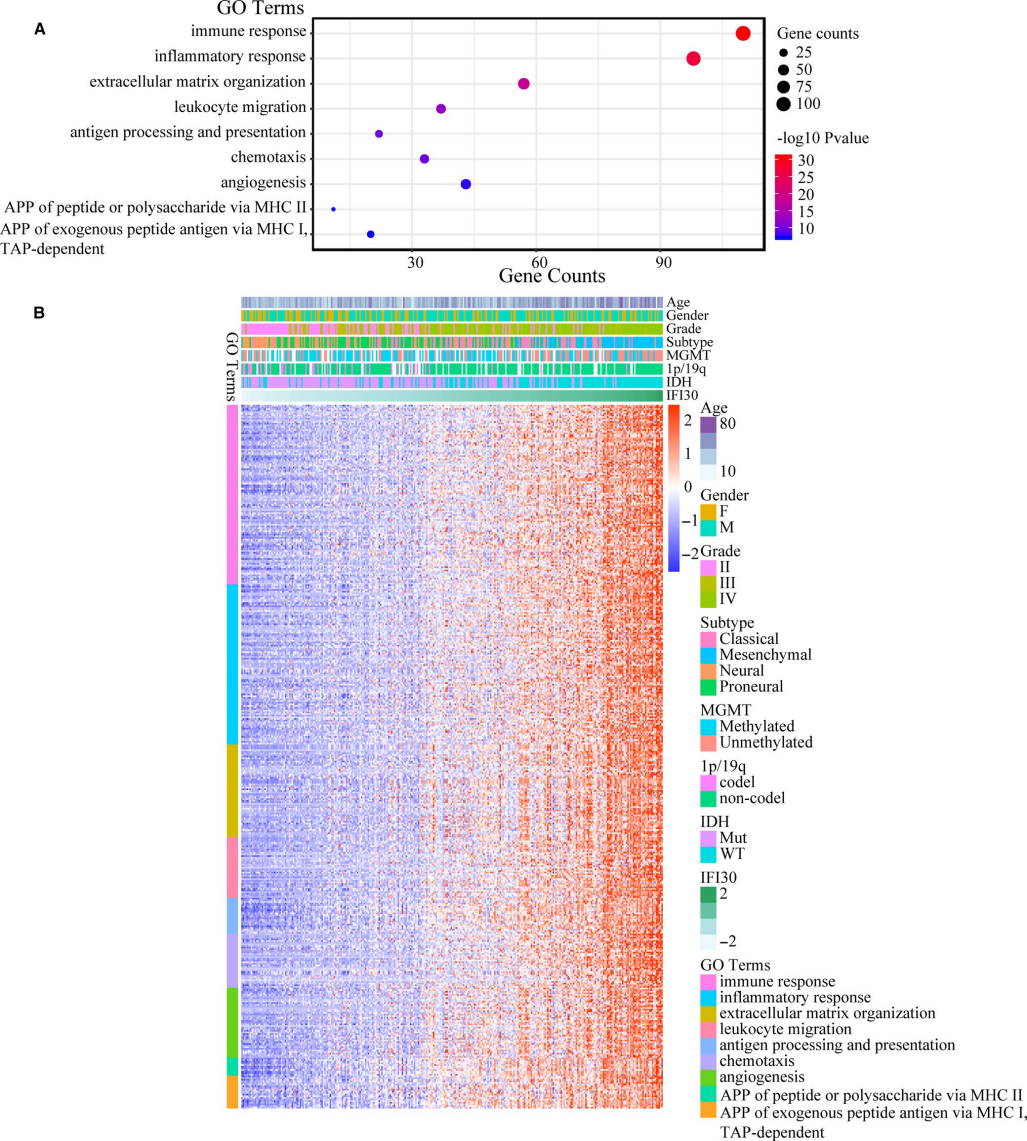
Functional analysis of *IFI30*‐related genes in the CGGA data set. (A) Enriched pathways of genes positively correlated with *IFI30*. (B) Heatmap of genes positively correlated with *IFI30*. APP, antigen processing and presentation; TAP, transporters associated with antigen processing

To determine the biological function of *IFI30* in gliomas, Pearson's correlation analysis was carried out to evaluate genes that are strongly correlated with *IFI30* (|R| ≥ 0.5, *P* < .01). The total numbers of positively related genes in the CGGA and TCGA data sets were 1485 and 2440, respectively. The two positively related gene sets were separately subjected to functional annotation analysis with DAVID, whereupon the genes were found to be involved mainly in the immune response, antigen processing and presentation, chemotaxis, extracellular matrix organization, and angiogenesis (Figure 5 for CGGA, Figure S5 for TCGA). Similarly, 1025 and 1633 negatively related genes in the CGGA and TCGA data sets, respectively, were screened and found to be annotated mainly to normal biological processes, such as chemical synaptic transmission, neurotransmitter secretion, and nervous system development (Figure S6 for CGGA, Figure S7 for TCGA). These results suggested that *IFI30* participated in antigen processing and presentation and the immune response, and its up‐regulation might promote the progression of gliomas via extracellular matrix organization and angiogenesis.

### 3.4 *IFI30* was associated with immunosuppressive phenotype in gliomas

**Figure 6:**
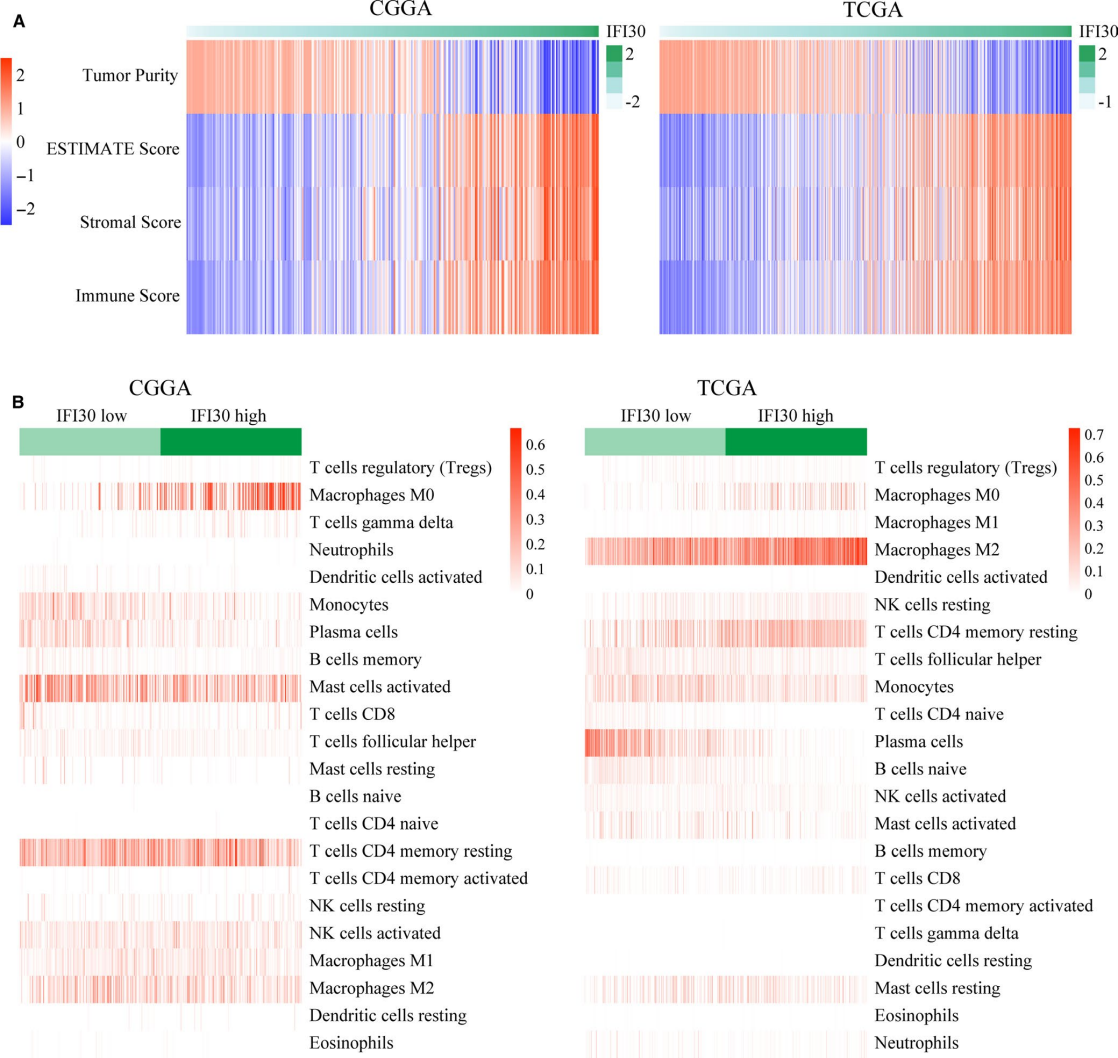
Association between *IFI30* expression and immune cell infiltration. (A) Positive correlation between *IFI30* expression and immune scores. (B) Immune cell infiltration in glioma samples with low and high *IFI30* expression levels

**Figure 7:**
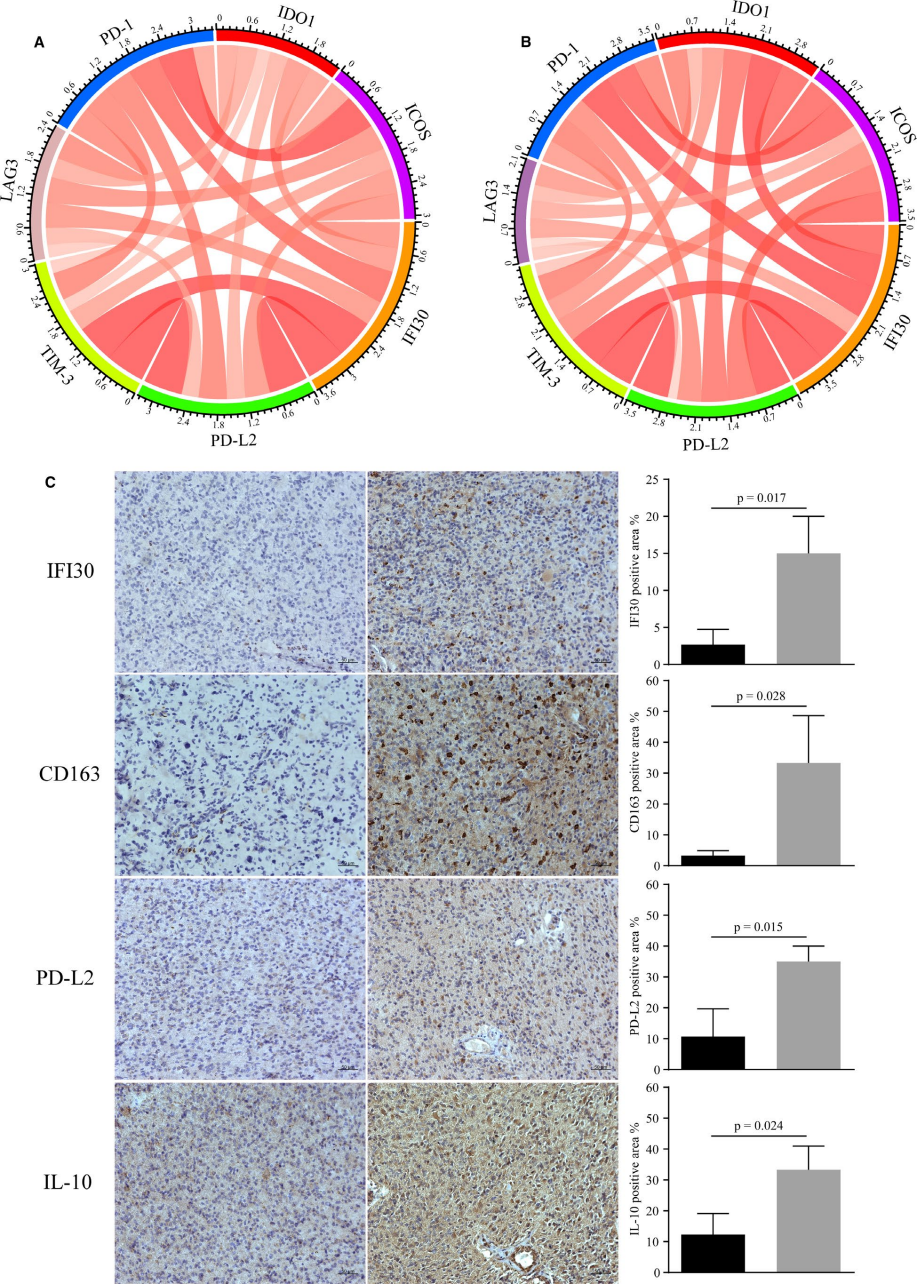
Positive correlation between *IFI30* expression and immune checkpoints in the CGGA (A) and TCGA (B) data sets. (C) Immunostaining of CD163, PD‐L2 and IL‐10 in glioma samples (n = 3) with low and high *IFI30* expression levels (bar, 50µm)

Given that *IFI30* participates in MHC II‐associated antigen processing, the discovery that its high expression implied poor outcomes for patients with gliomas prompted us to explore the relationship between *IFI30* expression and immune cell infiltration by applying the ESTIMATE algorithm.^33^ The immune scores increased with increase in the *IFI30* expression levels in both the CGGA and TCGA data sets (Figure 6A). Next, CIBERSORT was used to evaluate the abundance of various immune cell types in the CGGA and TCGA samples.^34^ As illustrated in Figure 6B, samples with high *IFI30* expression showed high numbers of immune cells, including regulatory T cells (Tregs), M0 macrophages, and gamma delta T cells in the data set from CGGA, and Tregs and M0, M1 and M2 macrophages in the data set from TCGA. Finally, we evaluated the correlation of *IFI30* expression with some important immune checkpoints, which could reflect the immune microenvironment of gliomas with different *IFI30* expression levels. As shown in Figure 7A,B, *IFI30* expression was significantly positively correlated with molecules that suppress the antitumour immune response, including PD‐1, PD‐L2, T cell immunoglobulin and mucin domain‐3 (TIM‐3), lymphocyte activation gene‐3 (LAG3),indoleamine 2,3‐dioxygenase 1 (IDO1) and inducible T cell costimulatory (ICOS).^35^, ^36^ To confirm the association between *IFI30* expression and the immune phenotype, immunohistochemical staining of CD163, PD‐L2, and IL‐10 was carried out on the glioma samples with low and high *IFI30* expression levels.^22^, ^23^ As shown in Figure 7C, the samples with high *IFI30* expression had higher levels of CD163, PD‐L2, and IL‐10 staining, which indicated an immunosuppressive microenvironment. These results might partially explain the poor outcome of patients with gliomas with a high level of *IFI30* expression.

## 4 DISCUSSION

According to the WHO classification of tumours of the CNS, gliomas—the most common primary intracranial tumours—can be classified into grade I‐IV on the basis of their histology and malignancy.^15^, ^37^ Grade I gliomas have a more circumscribed growth pattern and lower proliferative potential, whereas the grade II and III are generally infiltrative.^38^ Grade IV, also called glioblastoma, is the most malignant and most common subgroup of gliomas. In this study, we collected the mRNA sequencing data of 921 glioma samples from the CGGA and TCGA databases and analysed the expression pattern, prognostic value, and potential biological significance of the *IFI30* gene. In assessing the survival of patients with gliomas, only OS was analysed for the lack of enough data to support progression‐free survival analysis.

Published research studies on *IFI30*, the gene coding for the enzyme that is functionally associated with antigen processing, have been mainly performed on melanoma, breast cancer, and diffuse large B‐cell lymphoma (DLBCL). It has been reported that the absence of GILT in MHC II‐positive melanomas results in a deficiency in antigen processing and may contribute to the induction of immune unresponsiveness. Moreover, the transfection of melanoma cells with the GILT‐encoding gene enhanced the presentation of antigenic epitope.^39^, ^40^GILT expression is significantly decreased in both primary and metastatic breast cancer cells compared with that in normal epithelial cells. Breast cancers with reduced GILT expression have poor disease‐free survival.^41^ The association of low GILT expression with poor survival has also been validated in patients with DLBCL.^42^ These reports might seem contradictory to the results in gliomas, where high *IFI30* expression indicated poor OS, but this may be due to the specific immune microenvironment of gliomas. Our results showed that gliomas with higher *IFI30* expression were more infiltrated by M0 macrophages and Tregs, whereas their infiltration by CD8 T cells did not increase correspondingly. A recent study demonstrated that GBM‐associated myeloid cells resembled the M0 macrophage phenotype, which is consistent with our results.^43^ Undifferentiated M0 macrophages can polarize into classically activated macrophages (M1) with the pro‐inflammatory/antitumoural phenotype and can also polarize into alternatively activated macrophages (M2) with the immune‐suppressive/protumoural phenotype.^44^ Gliomas have long been reported to be infiltrated by macrophages.^45^ Glioma cells release several factors, such as colony‐stimulating factor 1 (CSF‐1), glial‐derived neurotrophic factor and granulocyte‐macrophage colony‐stimulating factor, to attract TAMs to the tumour site.^46^, ^47^, ^48^ However, TAMs secrete a wide array of cytokines, including epidermal growth factor and TGF‐β, to promote glioma migration and invasion.^46^, ^49^ Hence, the reduction of M2 macrophage polarization or the promotion of M1 macrophage polarization is a promising therapy in cancer treatment, and studies on this have been conducted in gliomas.^50^ For example, M1‐like macrophages combined with immune checkpoint antibodies could eradicate GBM in mouse models.^51^ CSF‐1R inhibition reduced M2 macrophage polarization and regressed established gliomas.^52^ Chlorogenic acid repolarized macrophages from the M2 to the M1 phenotype and reduced glioma growth.^53^ All these research reports underscore the possibilities and potentials of targeting TAMs in glioma treatment, and our results imply that patients with high *IFI30* expression might benefit most from such therapy.

In summary, we have conducted a comprehensive research study on *IFI30* expression in gliomas and ascertained through bioinformatic profiling that this gene would be an unfavourable prognostic predictor of this disease. The underlying molecular mechanisms involved and more applications of our findings in clinical practice should be further explored.

## CONFLICT OF INTEREST

The authors confirm that there are no conflicts of interest.

## AUTHORS CONTRIBUTION

XL analysed the data and wrote the paper, CS wrote the paper, SY and QJ contributed to the statistical analyses, FC revised the paper, and WL approved the submitted and final version.

## Supporting information

Fig S1

Fig S2

Fig S3

Fig S4

Fig S5

Fig S6

Fig S7

Table S1

## Data Availability

The data that support the findings of this study are available in the CGGA and TCGA repositories.
