# Supplementary figures and images for: IFI30 expression is an independent unfavourable prognostic factor in glioma
Source: J Cell Mol Med. 2020 Sep 23;24(21):12433–43. doi: 10.1111/jcmm.15758 (PMC7686962; doi:10.1111/jcmm.15758)

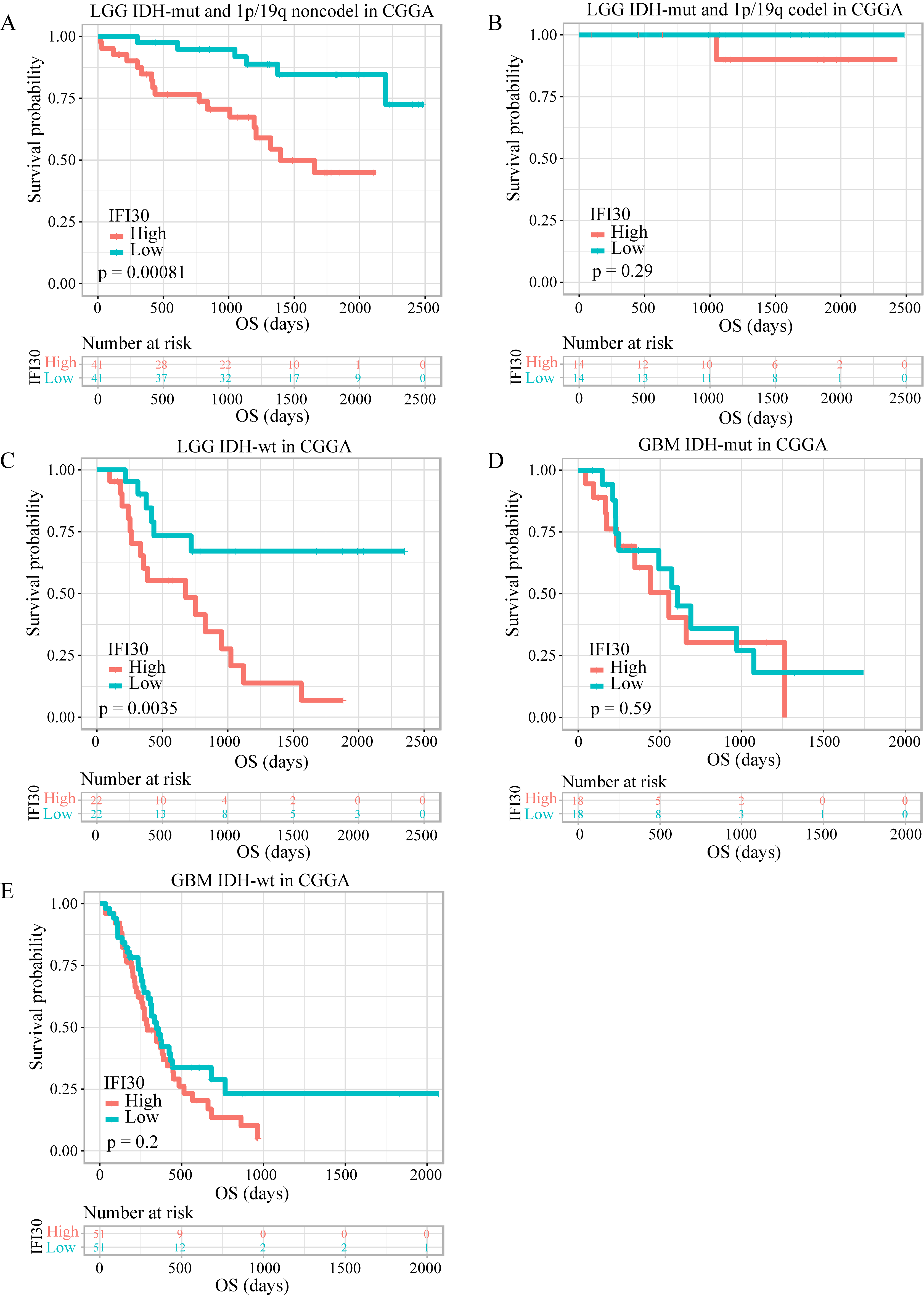

Supplement: Supplementary file 1 — Fig S1 [file JCMM-24-12433-s001.tif]

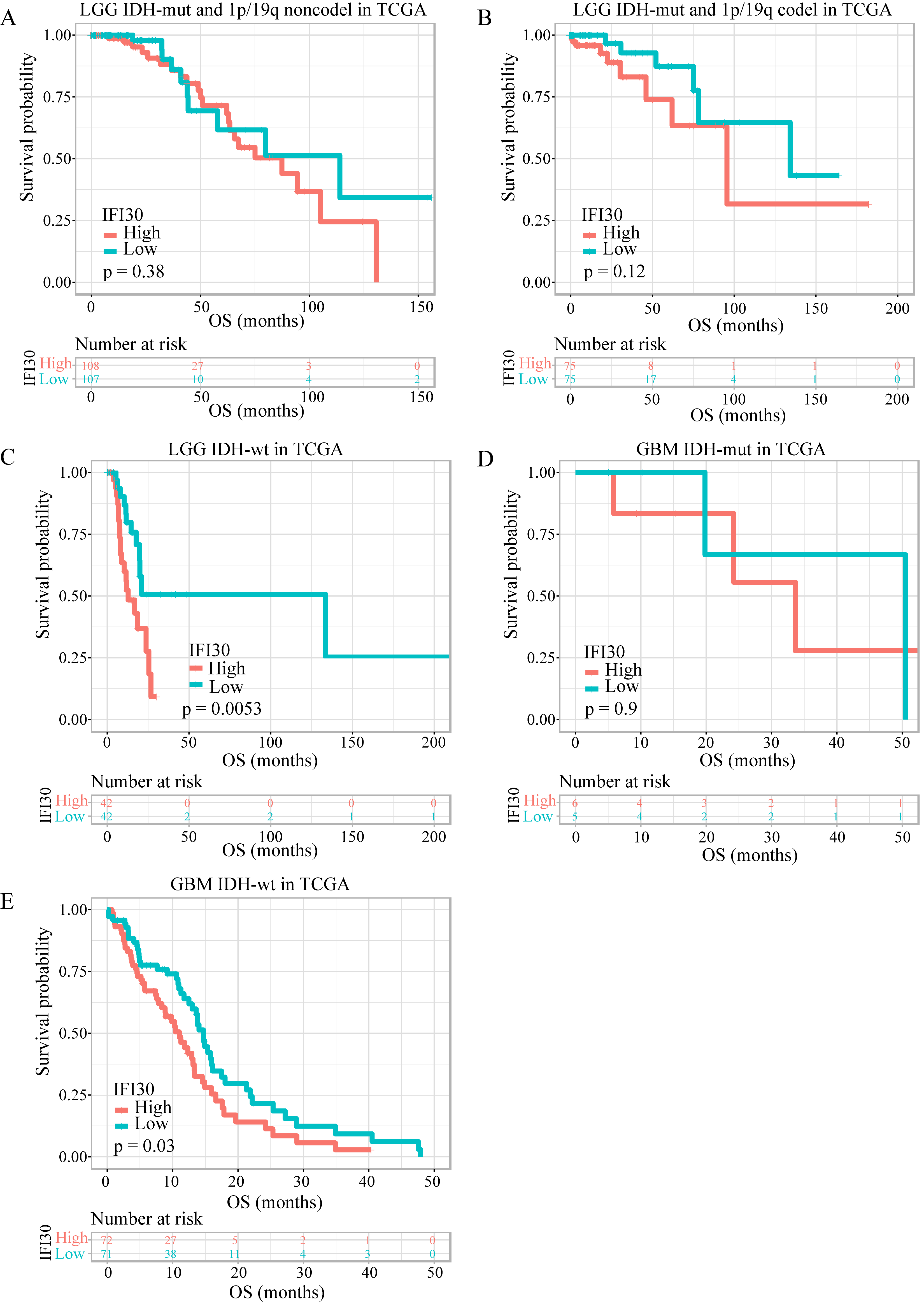

Supplement: Supplementary file 2 — Fig S2 [file JCMM-24-12433-s002.tif]

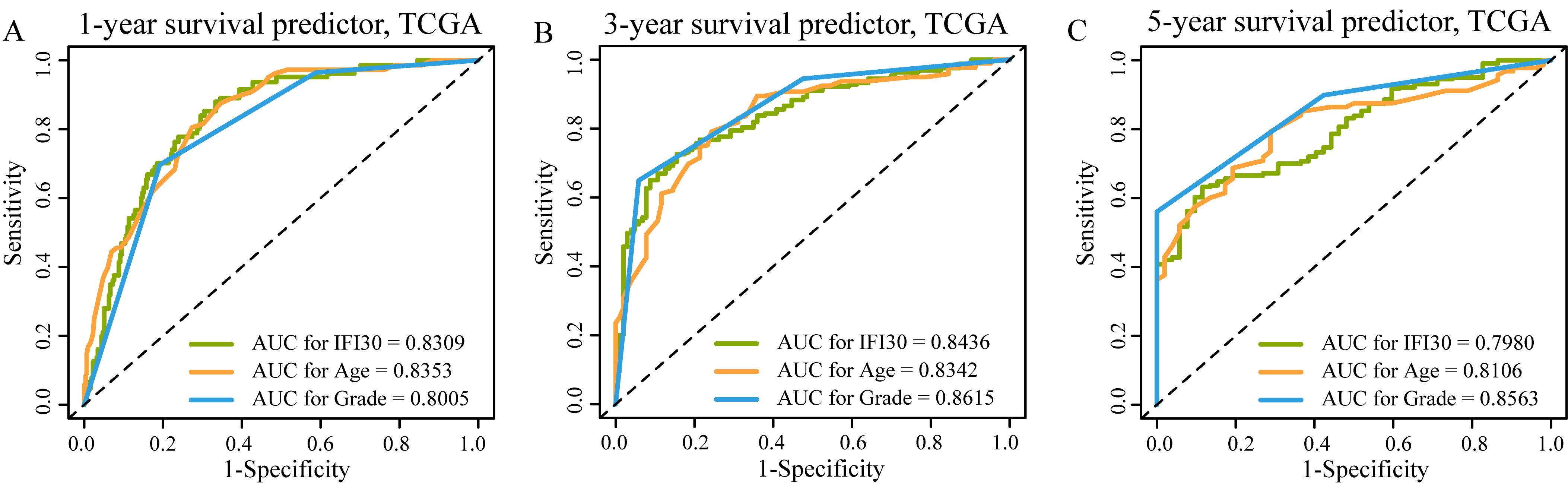

Supplement: Supplementary file 3 — Fig S3 [file JCMM-24-12433-s003.tif]

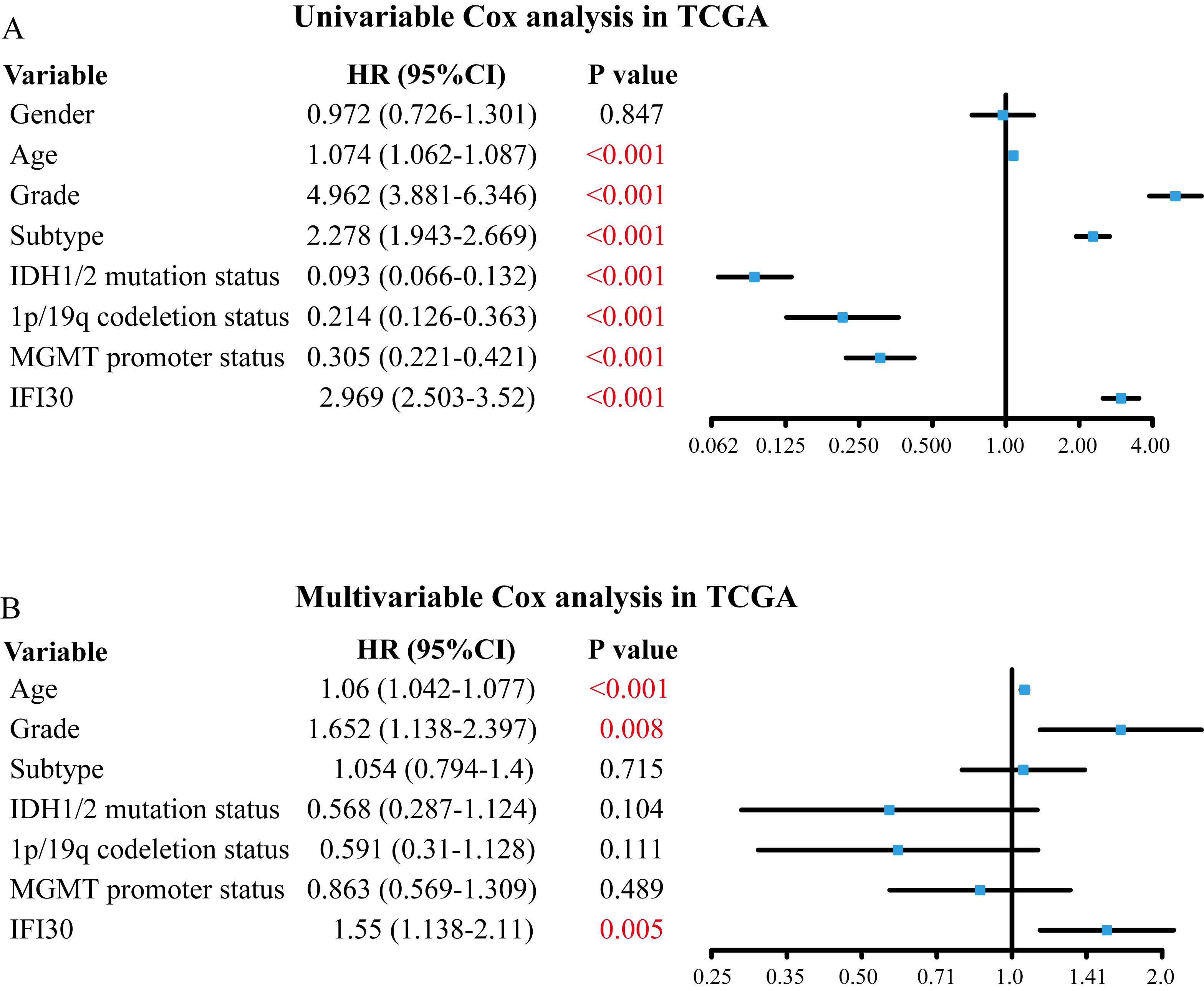

Supplement: Supplementary file 4 — Fig S4 [file JCMM-24-12433-s004.tif]

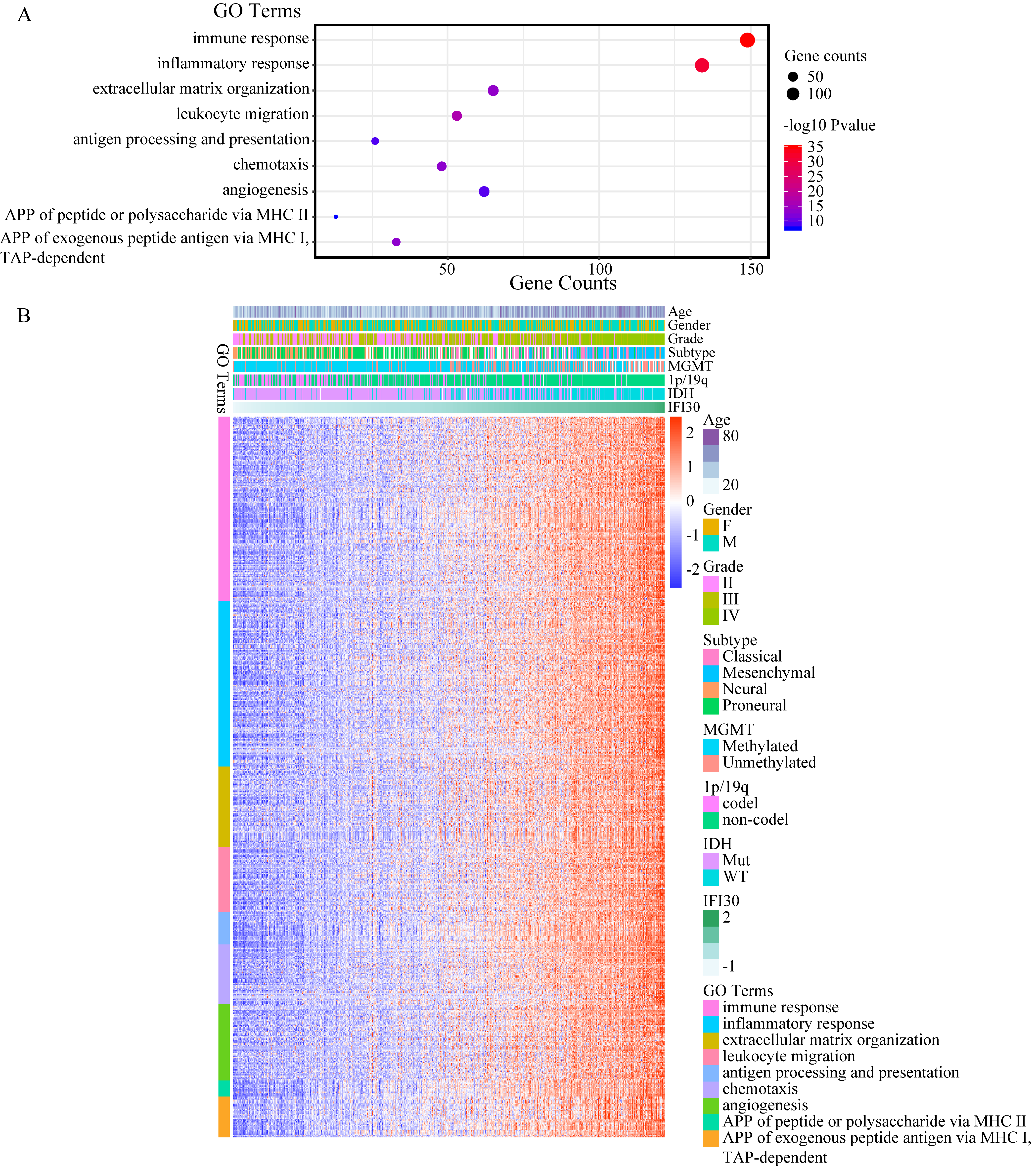

Supplement: Supplementary file 5 — Fig S5 [file JCMM-24-12433-s005.tif]

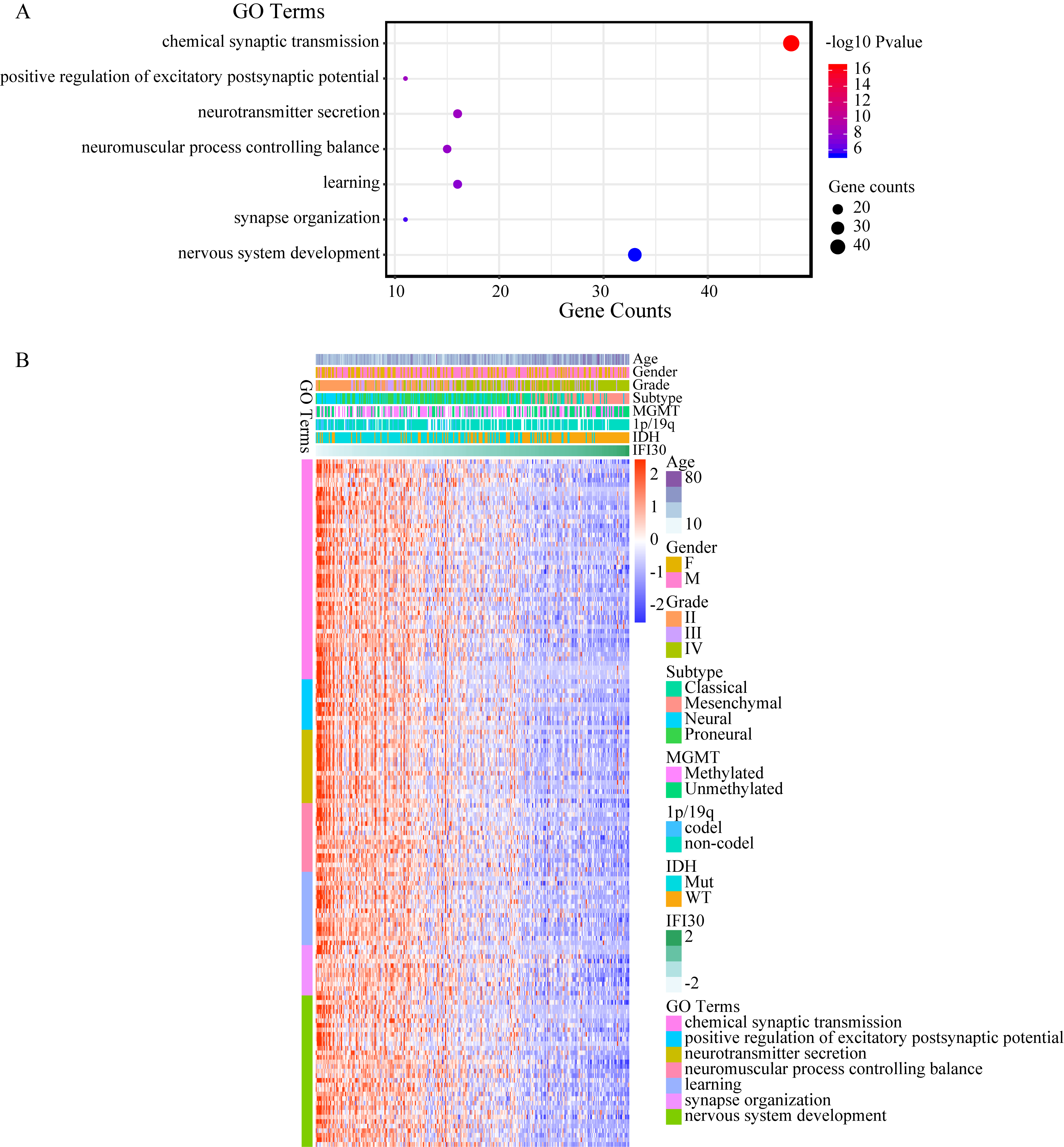

Supplement: Supplementary file 6 — Fig S6 [file JCMM-24-12433-s006.tif]

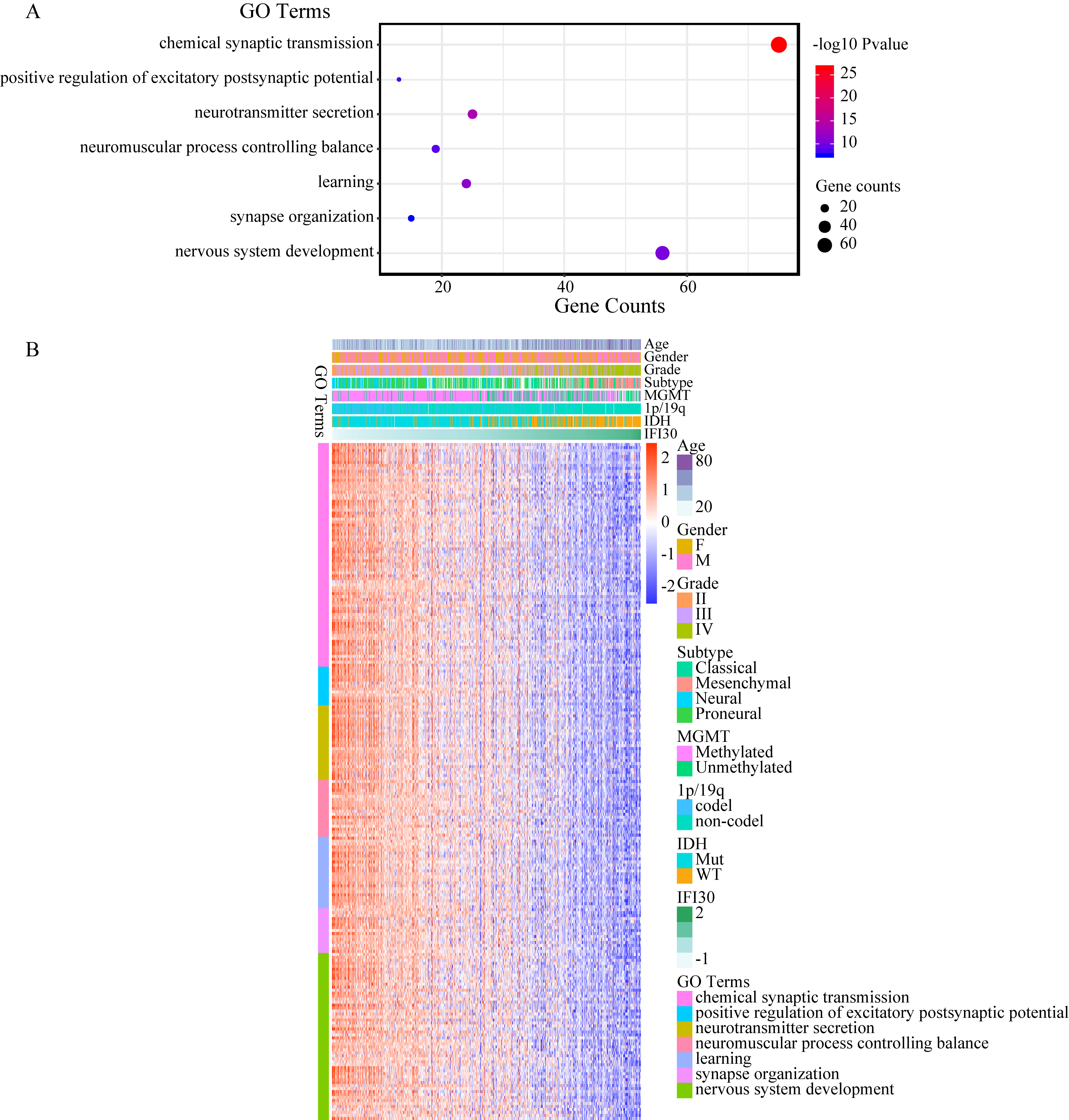

Supplement: Supplementary file 7 — Fig S7 [file JCMM-24-12433-s007.tif]
