# Supplementary material for: IFI30 expression is an independent unfavourable prognostic factor in glioma
Source: J Cell Mol Med. 2020 Sep 23;24(21):12433–43. doi: 10.1111/jcmm.15758 (PMC7686962; doi:10.1111/jcmm.15758)
Supplement: Supplementary file 8 — Table S1 [file JCMM-24-12433-s008.docx]

Table S1 Characteristics of glioma patients in CGGA and TCGA datasets

| Variable | CGGA  (n = 310) | TCGA  (n = 611) |
| --- | --- | --- |
| Median age (range) | 43 (8-81) | 47 (14-89) |
| Gender |  |  |
| Male | 195 | 357 |
| Female | 115 | 254 |
| Grade |  |  |
| II | 105 | 214 |
| III | 67 | 237 |
| IV | 138 | 160 |
| Subtype |  |  |
| Neural | 76 | 102 |
| Proneural | 99 | 218 |
| Classical | 70 | 84 |
| Mesenchymal | 65 | 94 |
| NA | 0 | 113 |
| IDH mutation status |  |  |
| WT | 146 | 227 |
| Mut | 164 | 376 |
| NA | 0 | 8 |
| 1p/19q codeletion status |  |  |
| No | 218 | 455 |
| Yes | 36 | 150 |
| NA | 56 | 6 |
| MGMT promoter status |  |  |
| Unmethylated | 110 | 151 |
| Methylated | 134 | 426 |
| NA | 66 | 34 |
| Radiotherapy |  |  |
| Yes | 207 | NA |
| No | 80 | NA |
| NA | 23 | NA |
| Chemotherapy |  |  |
| Yes | 155 | NA |
| No | 124 | NA |
| NA | 31 | NA |

CGGA: Chinese Glioma Genome Atlas; TCGA: The Cancer Genome Atlas; IDH: isocitrate dehydrogenase; MGMT: methylguanine methyltransferase; NA: not available
